# Focus on 16p13.3 Locus in Colon Cancer

**DOI:** 10.1371/journal.pone.0131421

**Published:** 2015-07-29

**Authors:** Evi Mampaey, Annelies Fieuw, Thalia Van Laethem, Liesbeth Ferdinande, Kathleen Claes, Wim Ceelen, Yves Van Nieuwenhove, Piet Pattyn, Marc De Man, Kim De Ruyck, Nadine Van Roy, Karen Geboes, Stéphanie Laurent

**Affiliations:** 1 Department of Gastroenterology–Digestive Oncology, Ghent University Hospital, Ghent, Belgium; 2 Department of Medical Genetics, Ghent University, Ghent, Belgium; 3 Department of Pathology, Ghent University Hospital, Ghent, Belgium; 4 Department of Gastrointestinal Surgery, Ghent University Hospital, Ghent, Belgium; 5 Department of Basic Medical Sciences, Ghent University, Ghent, Belgium; Deutsches Krebsforschungszentrum, GERMANY

## Abstract

**Background:**

With one million new cases of colorectal cancer (CRC) diagnosed annually in the world, CRC is the third most commonly diagnosed cancer in the Western world. Patients with stage I-III CRC can be cured with surgery but are at risk for recurrence. Colorectal cancer is characterized by the presence of chromosomal deletions and gains. Large genomic profiling studies have however not been conducted in this disease. The number of a specific genetic aberration in a tumour sample could correlate with recurrence-free survival or overall survival, possibly leading to its use as biomarker for therapeutic decisions. At this point there are not sufficient markers for prediction of disease recurrence in colorectal cancer, which can be used in the clinic to discriminate between stage II patients who will benefit from adjuvant chemotherapy. For instance, the benefit of adjuvant chemotherapy has been most clearly demonstrated in stage III disease with an approximately 30 percent relative reduction in the risk of disease recurrence. The benefits of adjuvant chemotherapy in stage II disease are less certain, the risk for relapse is much smaller in the overall group and the specific patients at risk are hard to identify.

**Materials and Methods:**

In this study, array-comparative genomic hybridization analysis (array-CGH) was applied to study high-resolution DNA copy number alterations in 93 colon carcinoma samples. These genomic data were combined with parameters like *KRAS* mutation status, microsatellite status and clinicopathological characteristics.

**Results:**

Both large and small chromosomal losses and gains were identified in our sample cohort. Recurrent gains were found for chromosome 1q, 7, 8q, 13 and 20 and losses were mostly found for 1p, 4, 8p, 14, 15, 17p, 18, 21 and 22. Data analysis demonstrated that loss of chromosome 4 is linked to a worse prognosis in our patients series. Besides these alterations, two interesting small regions of overlap were identified, which could be associated with disease recurrence. Gain of the 16p13.3 locus (including the RNA *binding protein*, *fox-1 homolog* gene, *RBFOX1*) was linked with a worse recurrence-free survival in our patient cohort. On the other hand, loss of *RBFOX1* was only found in patients without disease recurrence. Most interestingly, above mentioned characteristics were also found in stage II patients, for whom there is a high medical need for the identification of new prognostic biomarkers.

**Conclusions:**

In conclusion, copy number variation of the 16p13.3 locus seems to be an important parameter for prediction of disease recurrence in colon cancer.

## Introduction

With one million new cases of colorectal cancer (CRC) diagnosed annually in the world, CRC is the third most commonly diagnosed cancer in the Western world and the second leading cause of cancer-related deaths in both males and females (9% of all cancers) [1]. One of the challenges in CRC is to obtain a decrease in death rate by the implementation of screening programs leading to early detection and treatment of pre-cancerous lesions [2]. Therapeutic decisions are based on a staging system describing tumour extent, presence or absence of lymph node involvement and distant metastasis (TNM classification) [3]. Early stage CRC can be cured by surgery alone. The benefit of adjuvant chemotherapy has been most clearly demonstrated in stage III, whereas benefit in stage II disease remains controversial [3–5]. Nowadays stage III patients are probably overtreated: all patients receive chemotherapy while about half of the patients did not experience recurrence before the introduction of adjuvant treatment [6]. On the other hand, stage II patients are probably undertreated, since a substantial number of them will develop recurrent disease. Identification of high risk stage II patients who will benefit from adjuvant chemotherapy is highly needed but has thus far been proven difficult to demonstrate in clinical trials. The use of adjuvant therapy in stage II patients is based on a risk assessment evaluating a.o. microsatellite (MS) status, lymphovascular and perineural invasion, tumour differentiation and number of examined lymph nodes [6].

There is a need for the identification of additional (bio)markers for the prediction of relapse in this subgroup of patients. This will hopefully lead to the identification of patients that might benefit from adjuvant chemotherapy [3].

Recently, much progress has been made in the understanding of colorectal tumour biology. The histological sequence of colorectal carcinogenesis is characterized by several different genetic and epigenetic alterations [7,8], such as inactivating *APC* mutations in familial adenomatous polyposis (FAP) or the loss of function of the mismatch repair genes in hereditary non-polyposis colorectal cancer (HNPCC) [9,10]. In 1990, Fearon *et al*. suggested a cascade of mutations in different genes which is considered as the classical or chromosomal instability (CIN) pathway [7]. The first event in the early stages of a colorectal tumour is *APC* inactivation, followed over time by additional mutations of the *KRAS* and *TP53* genes, as well as deletions of chromosome 18q [7,11]. Most of the sporadic CRCs are characterized by those alterations and are localized in the left side of the colon. Besides this, two other pathways, involved in the etiology of CRC have been identified. Fifteen percent of sporadic CRCs and 95% of the HNPCC syndromes were shown to carry alterations in the microsatellite instability (MSI) pathway [10]. Finally, 20–30% of CRC have alterations of the serrated pathway, often presenting with a CpG-island methylated phenotype (CIMP) and/or methylation of the *MGMT* gene [12,13]. These (epi)genetic alterations can influence the course of the disease and thereby the outcome of the patients.

Another form of genetic alterations present in colorectal tumours are DNA copy number alterations (CNAs), which have already been studied in colon cancer, nevertheless without any consensus [14–17]. Due to the genomic instability of tumours, these CNAs are often present in tumours and represent potential new biomarkers for the prediction of disease recurrence.

In this study, CNAs were analysed in a large panel of colon cancer patients in different stages of disease with the aim to evaluate whether specific CNAs relate to patient outcome.

## Materials and Methods

### Patients and clinical data

A series of 159 primary colon adenocarcinoma resections were examined, prospectively collected between 2004 and 2012 from the department of Gastro-intestinal Surgery of the Ghent University Hospital in Belgium. The patients were followed until date of death or until clinical data cut-off (March 2014). The location of the tumour in the colon was designated as right for tumours located in the caecum, ascending colon and proximal half of the transverse colon. The location was designated as left when the tumour was located in the distal half of the transverse colon, descending colon and sigmoid. Analyses were performed within each tumor stage, counting from the date of diagnosis to relapse or date of last follow-up for disease recurrence (every stage without stage IV) and death or last follow-up for overall survival (all stages together). Whenever a correlation between survival and a certain marker was investigated over all tumour stages together, the relapsed patients were counted as stage IV patients and for these patients survival in this specific analysis was counted from the date of diagnosis of relapse.

Patients that were treated with adjuvant chemotherapy either received 5FU according to modified de Gramont (specifically stage II patients) or modified FOLFOX 6 (stage III patients) during 6 months. Four patients were treated in a clinical trial and may have received additional biological agents. Stage IV patients were treated with a variety of consecutive palliative regimens.

A written informed consent for participation was obtained from every patient. The study was approved by the commission for medical ethics of the Ghent University Hospital (Belgian registration number: B67020096625). Clinical data were retrieved from the medical files.

### Tissue samples

A pathologist, prior to formalin fixation, sampled both primary tumour and macroscopic normal tissues from resection specimens. Normal tissue was taken as far away as possible from the tumour in the same resection specimen. All samples were fresh frozen within a maximum of 30 minutes after surgical resection and stored at -80°C. A pathologist scored all tumour samples for tumour cell content on haematoxylin/eosin stained cryosections. Finally, 93 of 159 tumour samples containing at least 50% tumour cells were retained.

### KRAS mutation and MSI phenotype

All 159 tumour samples were profiled for the presence of seven somatic *KRAS* mutations in codon 12 and 13 (12ALA, 12ASP, 12VAL, 12CYS, 12SER, 12ARG and 13ASP) using the Therascreen KRAS RGQ PCR kit (Qiagen) on formalin-fixed paraffin-embedded (FFPE) tumour tissue (analysis under ISO15189 accreditation). Other *RAS* mutations were not investigated. The MS status of all 159 tumour samples was analysed by fragment analysis on the ABI3130xl genetic analyser (Applied Biosystems) using the GeneMapper software 4 (Applied Biosystems). BAT25, BAT26, D2S123, D17S250, NR21, D18S55, NR24 and NR27 are the eight investigated repeat markers. Tumours were classified as MSI when instability of 25% of the markers was observed. The MSI patients could be divided into two groups, MSI-high and MSI-low, according to the number of aberrant markers. However, the numbers were too low for proper statistical analysis. In situations where the distinction was made, it is disclosed in the text.

DNA was extracted using the DNeasy Blood and Tissue kit (Qiagen) according to the manufacturer’s instructions. The quality and quantity of the DNA samples were measured with a NanoDrop ND-1000 instrument (Thermo Scientific).

### Array comparative genomic hybridization

Copy number alterations were studied by oligo-based array-CGH on the tumour specimens with at least 50% tumour cells. Specimens with less tumour cells could bias the results. The samples were profiled on a Custom SurePrint G3 Human CGH Microarray, 4x180K (G4125A, Agilent Technologies). The DNA of the colon tumour samples was labeled with the fluorescent dye Cy3 (Perkin Elmer) and the same amount of commercially available reference male and female genomic DNA (Kreatech) was labeled with Cy5 (Perkin Elmer). Further processing was performed according to the manufacturer’s instructions (Agilent Technologies). Fluorescence intensities were measured using an Agilent scanner (G2505C, Agilent Technologies). Data were extracted using the Feature Extraction v10.1.1.1 software program (Agilent Technologies) and further processed with arrayCGHbase [18]. The cut-off values for gained and lost segments were 0.33 and -0.33 respectively. Aberrations that were present in more than 10 cases in a separately screened clinical genetics cohort of 1000 samples were considered as possible constitutional copy number variants (normal copy number alterations) and were excluded from further analysis. All reported results were manually validated.

### Statistics

The statistical environment R (version 2.12.1) was used to execute hierarchical cluster and heatmap analysis and to construct the graph showing the genome wide frequencies of all amplifications, gains and losses in all the samples. All other statistical analyses were performed in SPSS Statistics (version 21). (*) P<0.05, (**) p<0.01 and (***) p<0.001 were considered as statistically significant in two-tailed tests. Since all the data were normally distributed, ANOVA was used to compare populations and the Pearson correlation was used for the association between variables. Survival analyses were performed with the Kaplan-Meier method and the log rank test. Cox regression analysis was used to calculate hazard ratios.

## Results

### Clinical characteristics

The clinical data are summarized in S1 Table. All the tumours in this study were primary colon adenocarcinomas, which were untreated at the time of resection. The patients were predominantly from Caucasian ethnicity.

In terms of gender association, disease classification, site of the primary tumour, stage, differentiation, perineural and lymphovascular invasion, this set of 159 tumours closely resembles most other reported patient cohorts of sporadic CRC (S1 Table).

The tumours were equally distributed between men and women (57.9% vs 42.1%). The mucinous variant of adenocarcinoma was diagnosed in 20.1% of the tumours. All patients were staged using the AJCC-7 guidelines [19]. The patients were equally distributed over the four stages, with slightly more patients in stage II (36.5%). Half of the patients (49.7%) received adjuvant (31.2%) or palliative (18.5%) chemotherapy. At the end of follow-up, 105 patients (66%) were still alive (S1 Table). Among the 159 patients included in this study, 68 (52.7%) had an increased carcinoembryonic antigen (CEA) level at time of diagnosis. They had a poorer prognosis than those with a level below 3.4 ng/μl. Both metastatic or local recurrence and 2-year overall survival (OS) were significantly better for patients with a low CEA level at diagnosis (R = 0.260, **P = 0.003 for relapse-free survival (RFS); R = 0.271, **P = 0.002 for 2-year OS) (S1 File).

### KRAS mutation status and MS status and their correlations with the clinicopathological data

As shown in S1 Table, *KRAS*-mutations were detected in 51 patients (33.1%). Eight of these mutations were located in codon 13 (pGly13Asp), all others were located in codon 12 of the *KRAS* gene. Among the 159 patients tested for MSI, 87.9% of the colon cancers showed microsatellite stability (MSS), two patients (1.3%) were MSI-low and 16 patients (10.7%) were MSI-high (S1 Table).

In our patient population, we observed a significant correlation between the *KRAS* mutation and the MS status (R = -0.198, *P = 0.017). *KRAS* mutation frequency was higher in MSS patients than in MSI patients (S2 Table).

We found a significant correlation between the MS status with lymphovascular invasion (R = -0.181, *P = 0.038) and the site of the primary tumour (R = -0.291, ***P<0.001). We found MSI at least in one patient in every stage, but mostly in the early stages (two MSI patients in stage I, seven in stage II and one MSI patient both in stage III and IV) (Table 1). A significant correlation was found between MS status and tumour classification (R = 0.173, *P = 0.034) (S2 Table). This means that patients with MSI usually have a small tumour located in the right colon, in this cohort most often stage II mucinous adenocarcinoma without lymphovascular invasion. The colorectal tumours with MSI mostly are *KRAS* wild type (S2 File).

**Table 1:** Patient characteristics of the patient population with at least 50% of tumour cells (n = 93). An extended table of patient characteristics can be found in S1 and S2 Tables.

		Tumour stage			
		I (n = 17)	II (n = 37)	III (n = 20)	IV (n = 19)
**MS-status**	**MSS**	15 (88,2%)	28 (75,7%)	19 (95%)	15 (78,9%)
	**MSI-low**	-	-	-	1 (5,3%)
	**MSI-high**	2 (11,8%)	7 (18,9%)	1 (5%)	-
**Recurrence**	**Yes**	3 (17,6%)	3 (8,1%)	5 (25%)	-
	**No**	14 (82,4%)	33 (89,2%)	15 (75%)	-
	**Palliative**	-	-	-	19 (100%)
**2y-overall survival**	**Death**	-	5 (13,5%)	5 (25%)	9 (47,4%)
	**Alive**	17 (100%)	32 (86,5%)	15 (75%)	10 (52,6%)
**Lymphovascular invasion (Lv)**	**Present**	1 (5,9%)	5 (13,5%)	9 (45%)	15 (78,9%)
	**Absent**	14 (82,4%)	30 (81,1%)	8 (40%)	3 (15,8%)
***KRAS***	**Wild-type**	15 (88,2%)	24 (64,9%)	16 (80%)	13 (68,4%)
	**Mutant**	2 (11,8%)	13 (35,1%)	4 (20%)	6 (31,6%)
**16p13.3 locus**	**No alteration**	11 (64,7%)	25 (67,6%)	14 (70%)	10 (52,6%)
	**Loss**	4 (23,5%)	9 (24,3%)	4 (20%)	6 (31,6%)
	**Gain**	2 (11,8%)	3 (8,1%)	2 (10%)	3 (15,8%)

### DNA copy number alterations

**Fig 1:**
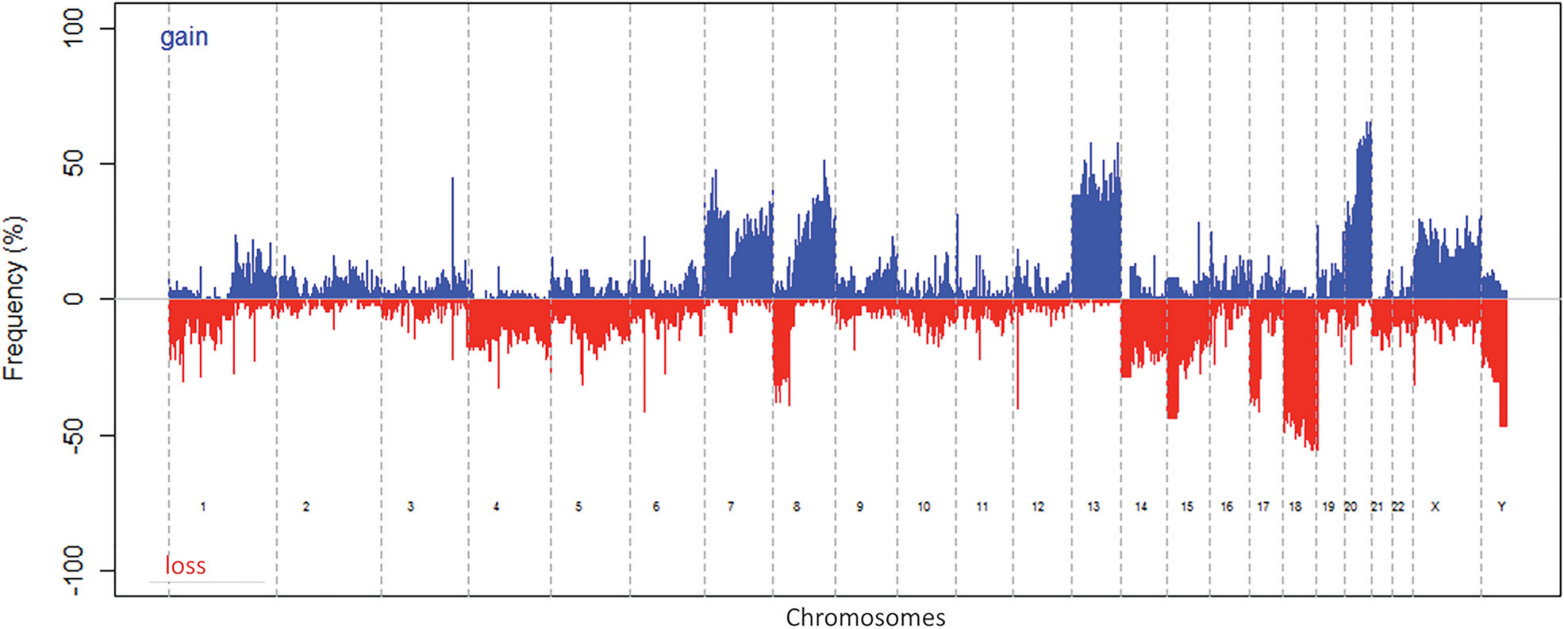
Frequency of DNA copy number alterations of all 93 samples for the whole genome. Gains are represented in blue, losses in red.

After scoring of all tumour samples, 93 samples with at least 50% of tumour cells were retained for array-CGH analysis. Ninety out of these 93 colon carcinomas showed genomic alterations (Fig 1). Patients with an increased CEA level had more CNAs than patients with a normal CEA level at diagnosis (*P = 0.023) (Figure A in S3 File). CNAs in our patient cohort were less abundant in MSI patients than in MSS patients, as evaluated by 180K array-CGH (*P = 0.016) (Figure B in S3 File). All the MSI patients had less than ten alterations, whereas the mean number of alterations for the overall patient cohort is 12.98 (± 0.982). When we looked for alterations in the different tumour stages, we found a higher number of alterations for the MSI patient only in stage IV, while in the other tumour stages, MSI patients had less CNAs compared to MSS patients (**P = 0.003) (Figure C in S3 File). Clustering analysis identified no statistically significant relationship between cluster groups (based on one or more specific alteration) and any clinical parameter in our database (S1 Fig).

The number of CNAs was divided in three groups after performing a ROC-curve (data not shown). Two cut-off points were established; the first one at 12 CNAs and the second one at 20 CNAs. In that way, we divided the colon carcinomas in three groups: tumours with less than 12 alterations, tumours with more than 20 alterations and an intermediate group of tumours with 12 till 20 alterations.

**Fig 2:**
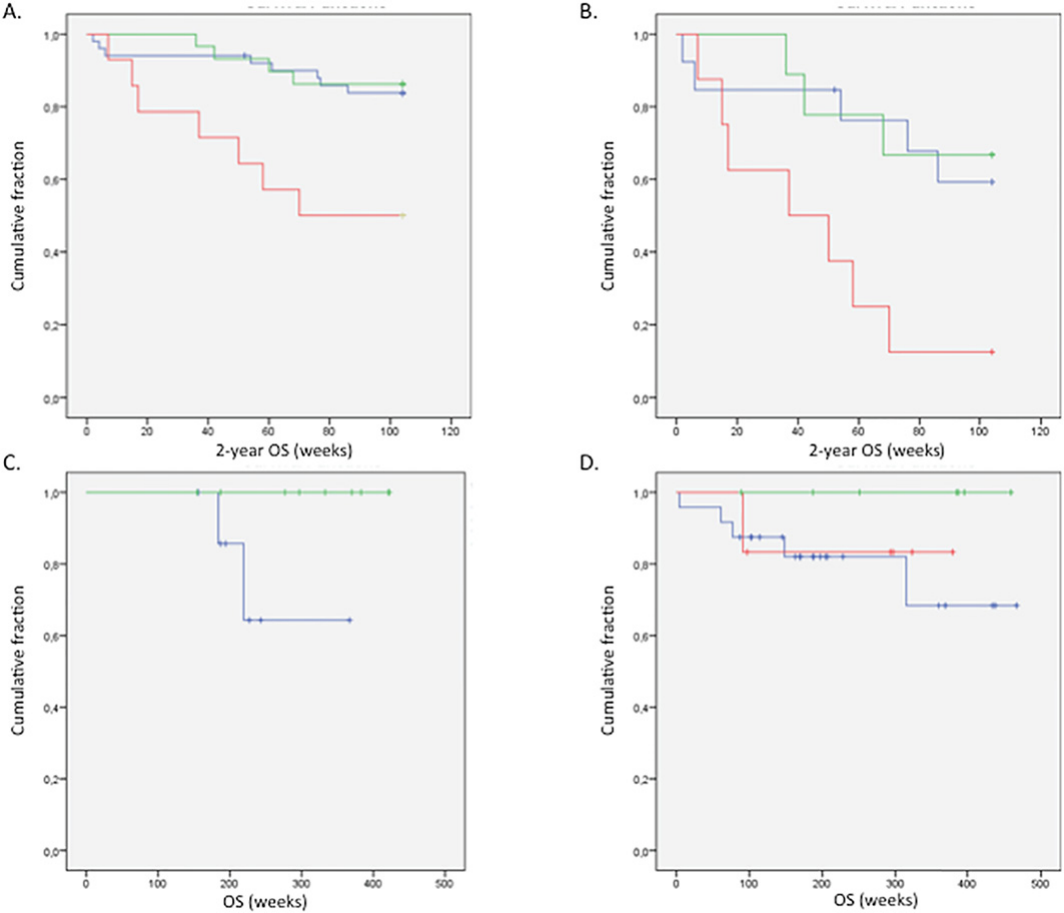
Kaplan Meier survival curves showing the number of CNAs and the overall survival. (A) For all stages, **P = 0,004; (B) for stage IV, *P = 0,015; (C) for stage I, *P = 0,019 and (D) for stage II, *P = 0,019. In the survival curve three groups can be distinguished; the group with less than twelve alterations (blue), the group with twelve untill twenty alterations (green) and the group with more than twenty alterations (red).

Survival analysis, calculated according to the number of alterations, revealed a shorter 2-year OS for the patients with more than 20 alterations in all tumour stages together (**P = 0.004) (Fig 2A). In patients with a stage IV tumour, the difference was remarkably larger (Fig 2B): the mean survival of patients with more than 20 alterations was half of that of patients with less than 20 alterations (*P = 0.015). Unexpectedly, in tumour stages I and II, poorer prognosis was found in the patient group with less than 12 alterations (*P = 0.019) (Fig 2C and 2D).

There is also a correlation between the differentiation grade of the tumour and the number of alterations. Patients, who have well differentiated tumours, had less than 12 CNAs, overall and for every stage separately. Patients with poorly differentiated tumours and with tumours carrying more than 20 CNAs, had a worse 2-year OS and specifically when we only compare stage IV patients (**P = 0.006 and **P = 0.010 respectively) (S2 Fig).

**Fig 3:**
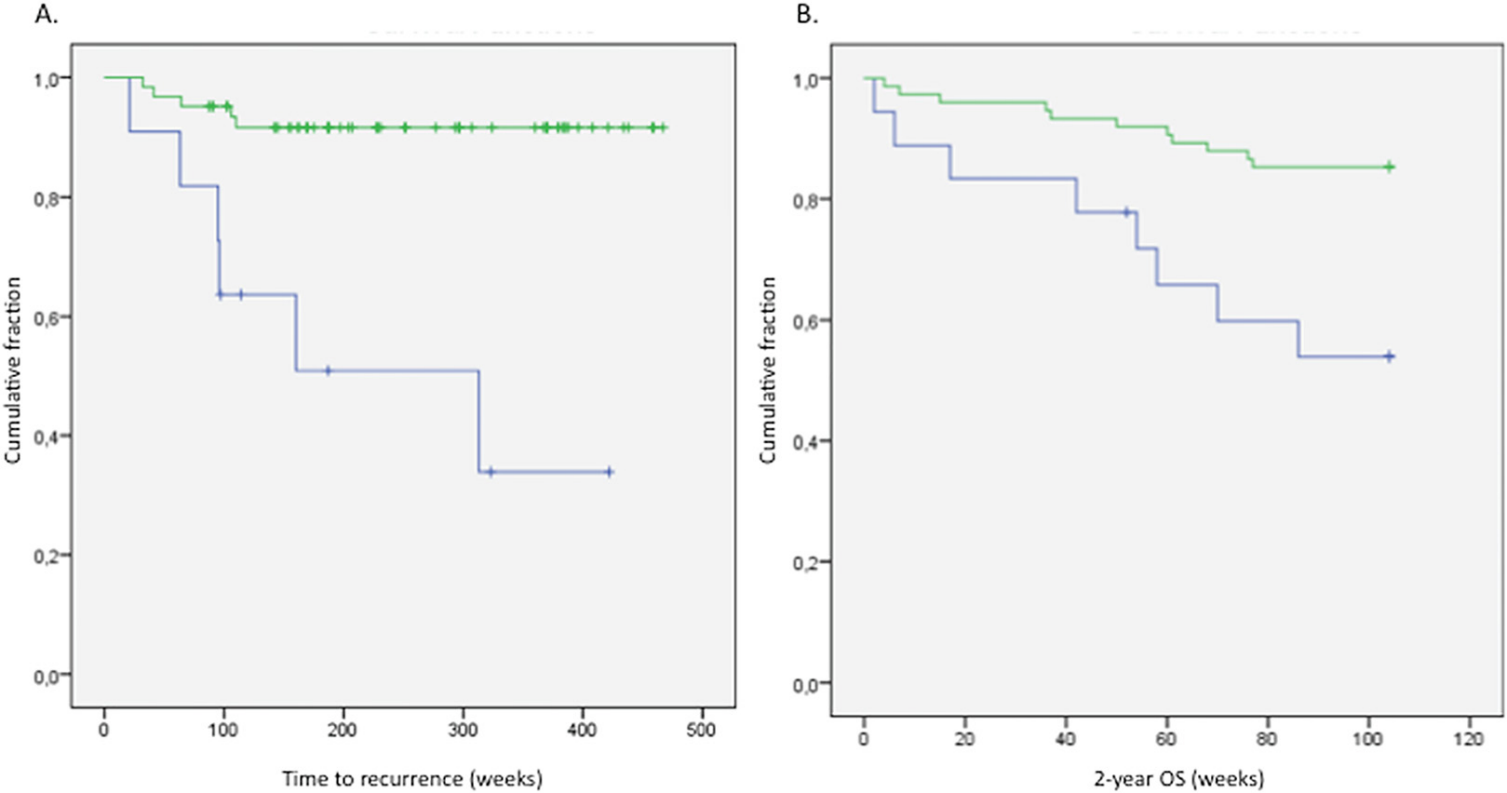
Kaplan Meier survival curves represented a worse prognosis for patients with loss of chromosome 4. Loss of chromosome 4 is linked with a shorter RFS, ***P<0,001 (A) and a smaller 2-year OS, **P = 0,002 (B). Patients without an alteration of chromosome 4 are presented in green, those with loss of chromosome 4 in blue.

Array comparative genomic hybridization (array-CGH) analysis showed that the most frequent CNAs in colon carcinomas were gains of chromosomes 1q, 7, 8q, 13, 20 and 20q and losses of chromosomes 1p, 4, 8p, 14, 15, 17p, 18, 21 and 22 (Fig 1). We also found the presence of multiple isochromosomes in our samples for chromosomes 1, 5 and 8 (respectively in 5.4%, 5.4% and 7.5% of our patients). Loss of chromosome 4 was significantly linked to a shorter RFS (mean RFS is 241.7 weeks for patients with a loss of chromosome 4 and 433.96 weeks for patients without chromosome 4 loss, ***P<0.001) (Fig 3A). Loss of chromosome 1, 3, 4 and 9 were linked to a shorter 2-year OS (respectively P = 0.053, **P = 0.005, **P = 0.002 and P = 0.069) (Fig 3B and Figures A-C in S4 File). Gain of chromosome 7 and 20 was shown to be significantly linked with a better 2-year OS (respectively *P = 0.048 and *P = 0.035) (Figures D-E in S4 File).

### Chromosome 16p13.3 alteration

Locus 16p13.3 was found to be a recurrent alteration, as it is present in 35% of our patient cohort of 93 patients. Eight patients showed gain of the distal part of this locus and 24 patients showed a loss with the most recurrent smallest region of overlap (SRO) from 6.5 till 7 Mb. Eight patients presented with a homozygous deletion. The SRO comprises exon 3 and 4 of the *RBFOX1* gene (6–7Mb). Copy number variant profiles of the normal tissue of the eight homozygous cases were evaluated to exclude a constitutional copy number variant. These results showed that the homozygous deletions were not constitutional copy number variants, but were somatically acquired alterations (S3 Fig). More than 70 genes, such as *CREBBP*, *MIR1225*, *RAB40C* and *RAB26*, are located in the distal part of the 16p13.3 locus. The latter two genes are members of the *RAS* oncogene family, which plays an important role in colon carcinogenesis.

**Fig 4:**
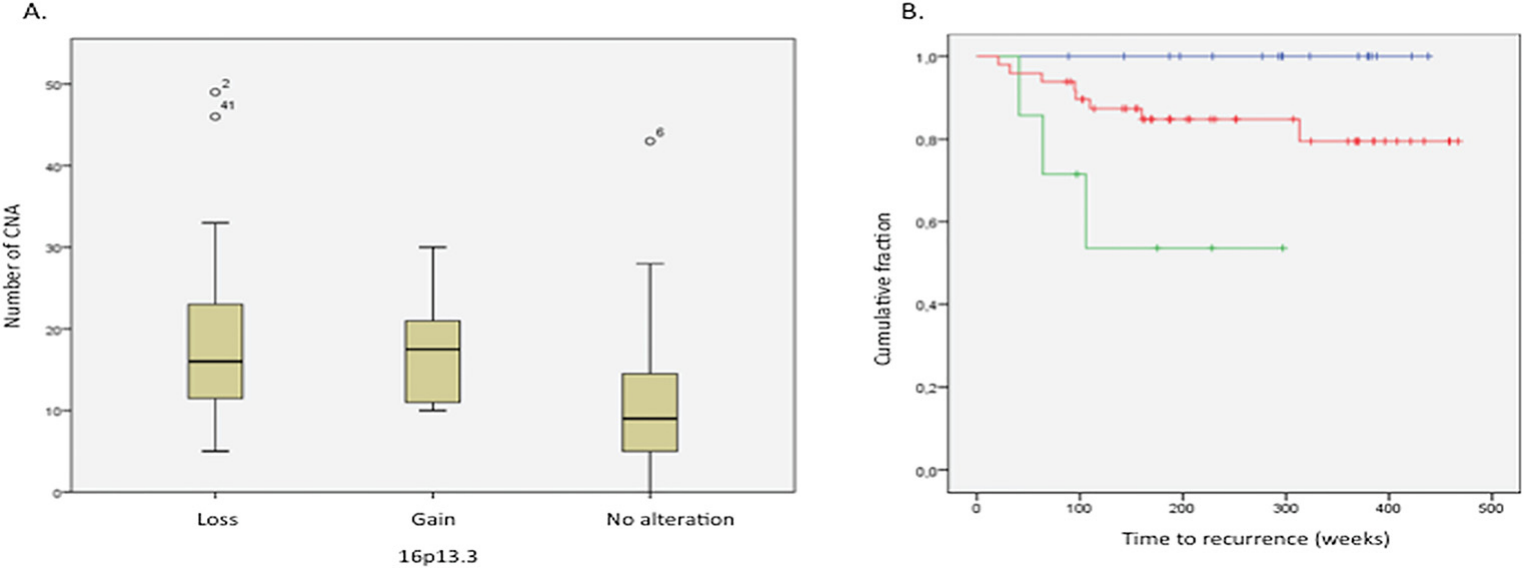
Gain of 16p13.3 is linked with a worse prognosis. (A) The relation of alteration of the 16p13.3 locus and the number of CNA in the corresponding patient for all tumour stages, R = -0,430 and ***P<0,001. (B) Kaplan Meier survival curve showing a worse prognosis for patients with a gain of the 16p13.3 locus over all tumour stages, **P = 0,010. Gains are represented in green, losses in blue and patients without an alteration are represented in red.

In general, patients without 16p13.3 alterations exhibit less CNAs (R = -0.430, ***P<0.001). This applies for all tumour stages (Fig 4A) as well as for every tumour stage separately (data not shown).

In stage IV tumours, a significant correlation was found between the presence of the 16p13.3 alteration (gain or loss) and the tumour in the right colon (R = -0.384, *P = 0.036). In all other tumour stages, this finding could not be confirmed, even not when all stages were considered together.

No correlation could be found between the 2-year OS and the presence of a 16p13.3 alteration (Figures A-D in S5 File). However, in the metastatic group, a trend was present (Figure E in S5 File). Patients who exhibited *RBFOX1* loss, showed a worse 2-year OS than patients without this deletion (P = 0.393).

Finally, a significant correlation was shown in all tumour stages between the presence of a 16p13.3 alteration and the RFS (Fig 4B and Figure A in S6 File). When a gain of 16p13.3 was present, the prognosis of these patients over all tumour stages was worse than the prognosis for patients without a 16p13.3 alteration or with a loss of the *RBFOX1* gene (HR = 5.018, **P = 0.020). When the same analysis was done for each tumour stage separately, a borderline significant correlation between the 16p13.3 alteration and disease recurrence was only observed in stage II tumours (*P = 0.022) (Figures B-D in S6 File). None of the patients who had a loss of the *RBFOX1* gene, suffered from a local or metastatic recurrence. On the contrary, patients without an alteration or with a gain in 16p13.3 had a higher risk of recurrence. The log-rank test for recurrence in stage II tumours showed a stronger significant correlation for gain of 16p13.3 and time of recurrence (**P = 0.008), with a mean time to recurrence of 166 weeks for patients with a gain of 16p13.3 and 443 weeks for patients without a 16p13.3 alteration (Figure E in S6 File).

## Discussion

The patient characteristics in this study largely correspond to those reported in literature. Some previously reported findings could however not be validated in this study, like a better prognosis for patients with MSI-high tumours as described by Gryfe and co-workers [20]. Colon tumours with a *KRAS* mutation are less likely to present microsatellite instability. The correlation between *KRAS* mutation and MS status has already been shown in previous studies and complicates the use of this marker in relation to prognosis [21–23].

This could be partly due to the fact that a real distinction of the MSI tumours in MSI-high and MSI-low could not be made. The association of MSI tumours with the histology of mucinous adenocarcinomas, which was described before, was confirmed in our patient population [24].

Our study questioned whether copy number alterations could be used as a prognostic marker for prediction of recurrence of colon cancer, especially in the stage II tumours. Using array-CGH analysis, we have identified recurrent CNAs among patients with colon carcinoma with different stages and clinical characteristics. The strengths of our study are the high number of patients and the integration of clinicopathological characteristics. Another strength is that we limited the study to colon cancer, because it has been suggested before that the large discrepancy in published studies is due to the fact that colon and rectal tumours were studied together [25]. In our study, 97% of the colon tumour samples possess genomic alterations. The fact that the number of CNAs is higher in stage II MSS tumours compared to MSI tumours has been suggested before and was validated by Brosens et al. [14]. Nakao and coworkers did also found a higher frequency of copy number alterations in the patients with MSS towards MSI-H (20% and 5% respectively). This was also true for gains, losses, amplifications and deletions separate [26]. Also the group of Xie found a lower CNA prevalence in MSI colorectal tumours than in MSS tumours [27]. The lower number of CNAs in MSI tumours could be an explanation for the better outcome of patients with an MSI tumour [20]. Our results confirm that patients with stage II tumours, and more specifically with pT3N0 tumours exhibiting high-frequency MSI, have a better prognosis. Their prognosis seems to be worse when treated with adjuvant chemotherapy based on 5-FU [28].

Gain of chromosome 13 and 20 and loss of chromosome 18 are the most common genomic alterations in colon cancer and those findings were confirmed in our study [29]. In addition, other recurrent gains and losses of whole-chromosomes were found. Gains on 1q, 7, 8q, 13, 20 and 20q and losses on 1p, 4, 8p, 14, 15, 17p, 18, 21 and 22 were mostly found. The occurrences of these CNAs are in agreement with reported data, but they were never reported all together in one study [15–17,25,30,31]. Loss of chromosome 1p and gain of chromosome 1q is most probably leading to the formation of an isochromosome, which is present in 5% of our patient population. In literature, there is very little information on formation of isochromosomes in CRC cell lines and even less in patient samples [15,32]. More information about the functionality of this isochromosome formation might be found by extending the patient cohort.

Statistical analysis showed that loss of chromosomes 1, 3, 4 and 9 was linked with a shorter survival. On the other hand, gain of chromosomes 7 and 20 is associated with a better 2-year OS. Moreover, we found a strong significant association for the loss of chromosome 4 and disease recurrence. Previous studies have shown, both by karyotyping and by meta-analysis of CGH studies, that loss of chromosome 4 is associated with poor clinical outcome [15,33]. More specifically, in stage II colon cancer patients, loss of chromosome 4q has already been associated with disease relapse [14]. Loss of chromosome 4 thus seems to be an important event in the etiology of colon cancer and could be used as a prognostic marker for recurrence in this disease.

Tumour genomes often possess many subchromosomal and focal alterations next to the alterations of complete chromosomes and chromosome arms [34,35]. The array-CGH data in this study revealed a recurrent altered locus, 16p13.3, which contains the *RBFOX1 (RNA binding protein*, *fox-1 homolog)* gene. This locus has not been reported as recurrent alteration with a clear role in the outcome in other array-CGH studies with an acceptable amount of patients with CRC [14–17,25,32,33]. Just a few studies have mentioned genetic alterations on 16p13.3 in CRC [27,30,31]. Our results confirm the latter studies. In our study, two SROs in this locus were linked with important clinical parameters. Gain of the distal 5Mb part of the locus was found in 9% of patients in this study. Loss of exon 3 and 4 of the *RBFOX1* gene was present in 26% of our patients, with the presence of a homozygous deletion of *RBFOX1* in one third of them. To rule out the possibility of a constitutional copy number variant, normal tissue was tested and found to be normal. Therefore, the 16p13.3 alteration can be classified as a somatic variant, only present in tumor cells.

Patients with a 16p13.3 alteration were found to exhibit more CNAs than patients without an alteration of the 16p13.3 locus. This holds for all tumour stages together as well as for each tumour stage separately. A gain in the 16p13.3 locus was associated with a worse RFS for all the stages together as well as for stage II tumours separately, which is a very interesting finding because of the high clinical need of new prognostic markers in stage II colon cancer. In different solid tumour types, like breast, lung, hepatocellular and pancreatic cancer gain of 16p13.3 was associated with late stage disease, worse survival and poor differentiation and a late stage disease [36–39]. Different genes located on the 16p13.3 locus were hypothesized to be implicated in the poor prognosis: in lung cancer *TSC2* was brought forward, in pancreatic cancer *PDPK1* [37,39]. The 16p13.3 locus bears a total of 167 known protein-coding genes of which *AXIN1*, *RAB40C* and *RAB26* are important genes in CRC. It had already been shown that *AXIN1* was mutated in colorectal cancers and wild-type can induce apoptosis in colorectal cancer cells [40]. The *RBFOX1* gene is also located in this region. Only few previous publications considering this gene in CRC were found. One specifically cites a high prevalence in a Bangladeshi population with early stage CRC [41]. Correlation with outcome was not investigated in this small patient group. Deletion of *RBFOX1* was also present in a significant proportion of CRC (106/419) in The Cancer Genome Atlas dataset [42].

Andersen *et al*. described a frequent loss, including homozygous loss, of the *RBFOX1* gene, associated with a poor clinical outcome in colon cancer [30]. We found a link between gain of the 16p13.3 locus and worse RFS. Moreover, no patients carrying a somatically acquired *RBFOX1* deletion showed a local or metastatic recurrence. It seems unlikely that an association between recurrence and loss of *RBFOX1* would be found, even after a longer follow-up. Moreover, the number of losses of *RBFOX1* is much higher than the number of gains of the 16p13.3 locus. *RBFOX1* alterations were also found to be associated with other diseases, like non-small-cell lung cancer, refractive error of the eye, mental disorders and neurodevelopmental disorders [43–46]. In neurodevelopmental and neuropsychiatric disorders losses of the same exons of *RBFOX1* were found [47]. Single nucleotide polymorphisms were found in *RBFOX1* in the region proximal to our SRO in colorectal adenomas [48]. The exact role of *RBFOX1* in diseases however, remains unclear, including its expression in the colon [41]. At present, it is only known that the *RBFOX1* gene codes for a tissue-specific alternative splicing protein [49,50]. Very little is known about the expression or role of *RBFOX1* in the intestine. Studies with immunohistochemistry have confirmed that *RBFOX1* is expressed at low levels in normal gut tissues and that expression is often lost in CRC [41]. Functionally, loss of *RBFOX1* activity may lead to aberrations in the splicing of a significant number of genes, generating diverse functional products that vary from those found in normal tissue. Alternative splicing is a key feature of cancer. *RBFOX1* encodes a ribonucleoprotein motif that is highly conserved among RNA-binding proteins; it binds to the C-terminus of *ataxin-2* and regulates alternative splicing of tissue-specific exons by binding to the hexanucleotide UGCAUG [51,52]. This could suggest an important basic function in development and differentiation for *RBFOX1* [51,53].

## Conclusions

In conclusion, the present array-CGH study was performed on a large and unbiased patient population consisting of different tumour stages and with a sufficiently high number of recurrences and deaths in all tumour stages. We could confirm that loss of chromosome 4 is linked with a worse prognosis in colon cancer. We identified gain of the 16p13.3 locus and loss of the *RBFOX1* gene as candidate biomarkers to predict recurrence in colon cancer; especially in stage II tumours where there is a high medical need for new prognostic markers. More interestingly, loss of *RBFOX1* is a marker excluding disease recurrence in colon cancer. Further study is needed to validate these results and to reveal the mechanisms by which these genomic alterations may be involved in colon carcinogenesis.

## Supporting Information

S1 Fig Heatmap of cluster analyses of the CNAs of the chromosomes and chromosome arms and the four different stages of colon cancer. Tumours are displayed as columns, grouped by the different stages of colon cancer as indicated by four different colours (green, blue, red and yellow). Chromosomes and chromosome arms used for this clustering are displayed as rows. As indicated in the color key; red blocks are losses and blue are gains for a full chromosome or chromosome arm.
(TIF)

S2 Fig Boxplot for overall survival (OS) in weeks grouped by the number of copy number alterations (CNAs) and the differentiation grade (moderate, poor, well or non-differentiated). In blue the patients with less than 12 CNAs, in green from 12 till 20 CNAs and in yellow the patients with more than 20 alterations.
(TIF)

S3 Fig DNA copy number profiles of the *RBFOX1* gene. DNA copy number profiles are shown for tumour (at the top) and normal (at the bottom) tissue from the same patient. There is a clear homozygous deletion in the *RBFOX1* gene in the tumour sample (red) and no alteration at all in the profile of the normal tissue.
(TIF)

S1 File Boxplots for recurrence free survival (Figure A) and 2-year overall survival (Figure B) in weeks grouped by CEA level at diagnosis. Tumour samples were categorized by their CEA level at diagnosis (more or less than 3,4ng/μl).
(TIF)

S2 File Effect of microsatellite status. Tumour stage (Figure A) and lymphovascular invasion (Figure B) for microsatellite stable (MSS) versus instability (MSI) patients with colon cancer.
(TIF)

S3 File Boxplots for copy number alterations (CNA) grouped by CEA level at diagnosis (Figure A) and MS status (Figure B). The CNAs are also grouped by MS status and tumour stage together (Figure C); with the MSS patients in blue and the MSI ones in green.
(TIF)

S4 File Kaplan-Meier survival curves according to the overall survival for different chromosome alterations. (Figure A) 2-year OS and chromosome 1 loss (blue = loss, green = no alteration); (Figure B) 2-year OS and chromosome 3 alteration (blue = loss, green = no alteration, yellow = gain); (Figure C) 2-year OS and chromosome 9 alteration (blue = loss, green = no alteration, yellow = gain); (Figure D) 2-year OS and chromosome 7 gain (blue = no alteration, green = gain); (Figure E) OS and chromosome 20 gain (blue = no alteration, green = gain).
(TIF)

S5 File 2-year overall survival (OS) curves estimated by the Kaplan-Meier method for all stages together (Figure A), stage I (Figure B), stage II (Figure C), stage III (Figure D) and stage IV (Figure E) in colon cancer comparing with an alteration in the 16p13.3 locus. In blue, patients with a loss, in green, patients with a gain and in yellow patients without an alteration in the 16p13.3 locus.
(TIF)

S6 File Recurrence free survival in association with the 16p13.3 locus. (Figure A) Boxplot for recurrence free survival in colon cancer and an alteration in the 16p13.3 locus (loss, gain or no alteration). Recurrence free survival (RFS) curves estimated by the Kaplan-Meier method for stage I (Figure B), stage II (Figure C), stage III (Figure D) and stage IV (Figure E) in colon cancer comparing with an alteration in the 16p13.3 locus. In blue, patients with a loss, in green, patients with a gain and in yellow patients without an alteration in the 16p13.3 locus.
(TIF)

S1 Table Clinicopathological data for 159 patients with colon cancer (tissues: colorectal tumour n = 159 and normal n = 159). (PDF)

S2 Table Pearson correlations showing the relationships between the different clinicopathological characteristics (n = 159). (PDF)
