# Supplementary figures and images for: Focus on 16p13.3 Locus in Colon Cancer
Source: PLoS One. 2015 Jul 29;10(7):e0131421. doi: 10.1371/journal.pone.0131421 (PMC4519182; doi:10.1371/journal.pone.0131421)

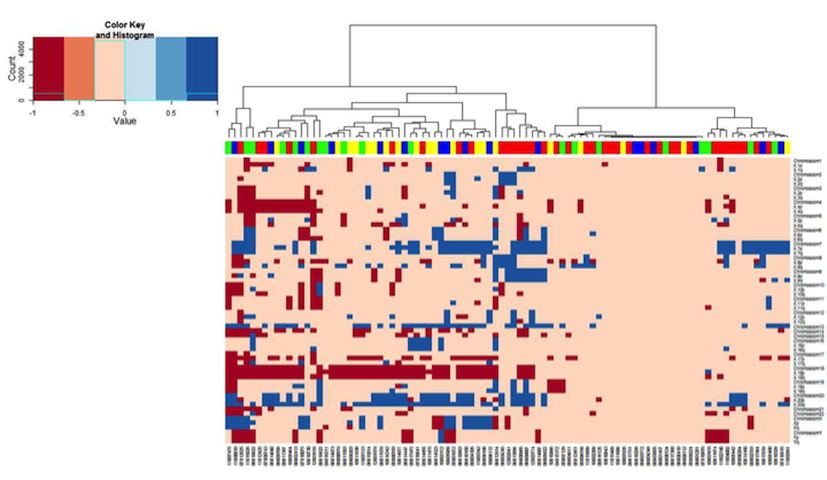

Supplement: S1 Fig — Tumours are displayed as columns, grouped by the different stages of colon cancer as indicated by four different colours (green, blue, red and yellow). Chromosomes and chromosome arms used for this clustering are displayed as rows. As indicated in the color key; red blocks are losses and blue are gains for a full chromosome or chromosome arm. (TIF) [file pone.0131421.s001.tif]

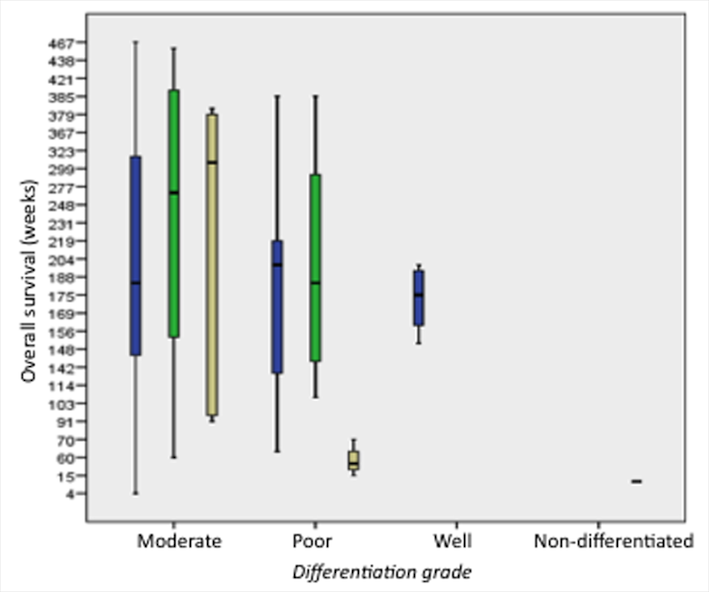

Supplement: S2 Fig — In blue the patients with less than 12 CNAs, in green from 12 till 20 CNAs and in yellow the patients with more than 20 alterations. (TIF) [file pone.0131421.s002.tif]

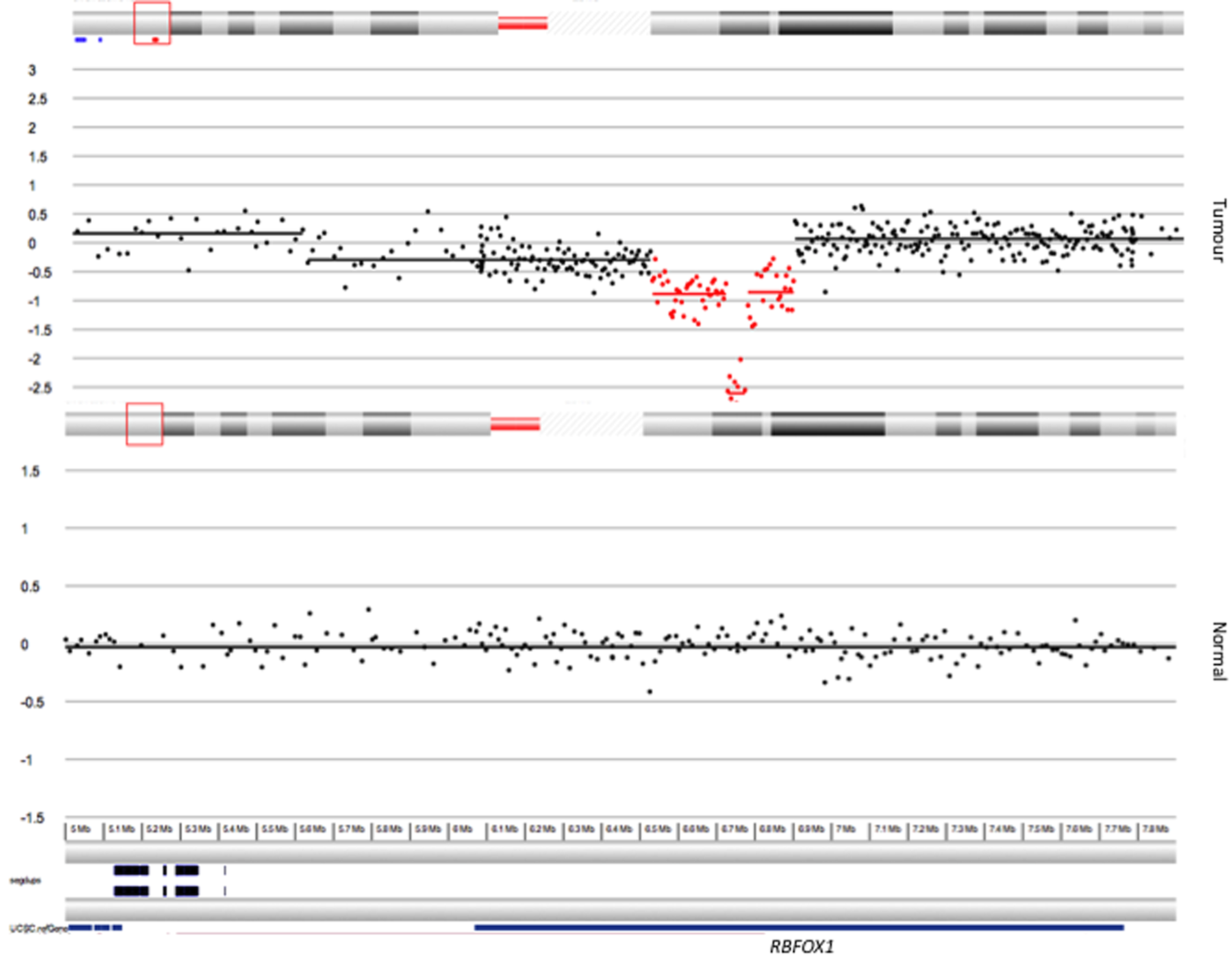

Supplement: S3 Fig — DNA copy number profiles are shown for tumour (at the top) and normal (at the bottom) tissue from the same patient. There is a clear homozygous deletion in the RBFOX1 gene in the tumour sample (red) and no alteration at all in the profile of the normal tissue. (TIF) [file pone.0131421.s003.tif]

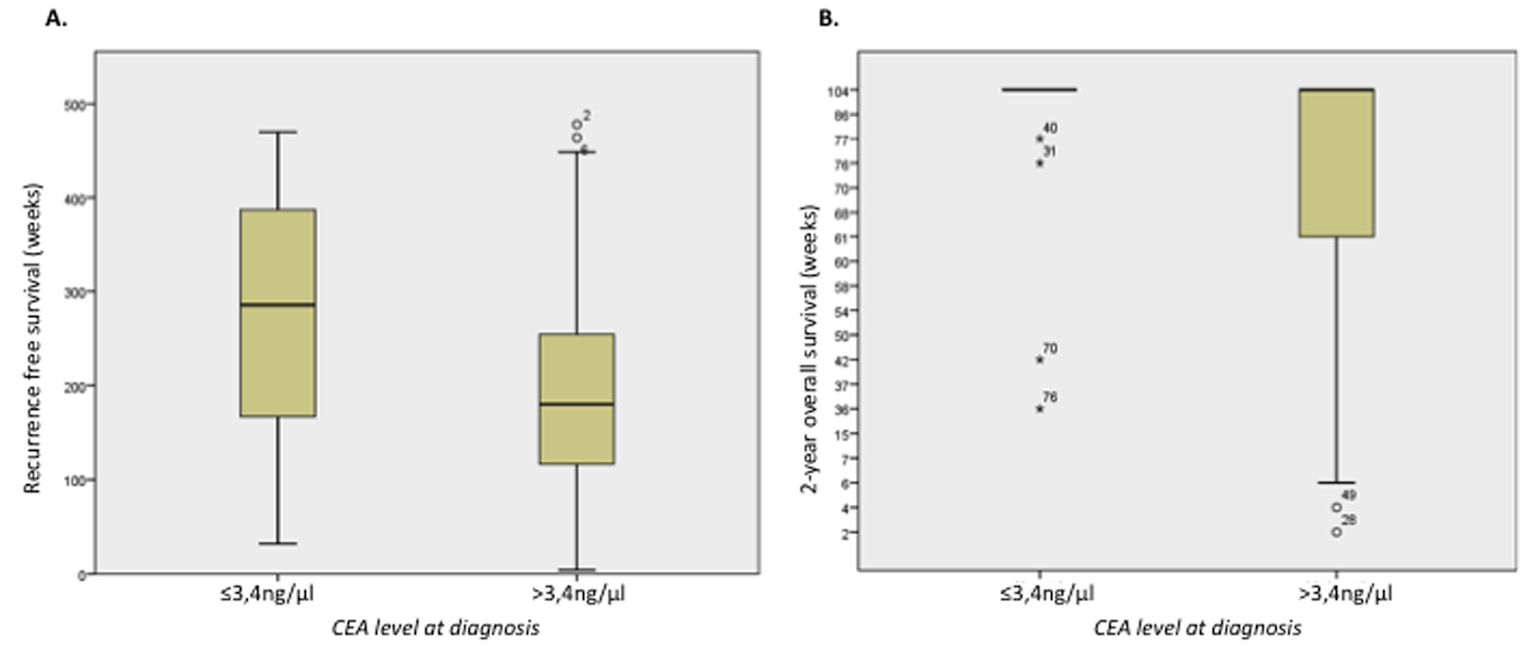

Supplement: S1 File — Tumour samples were categorized by their CEA level at diagnosis (more or less than 3,4ng/μl). (TIF) [file pone.0131421.s004.tif]

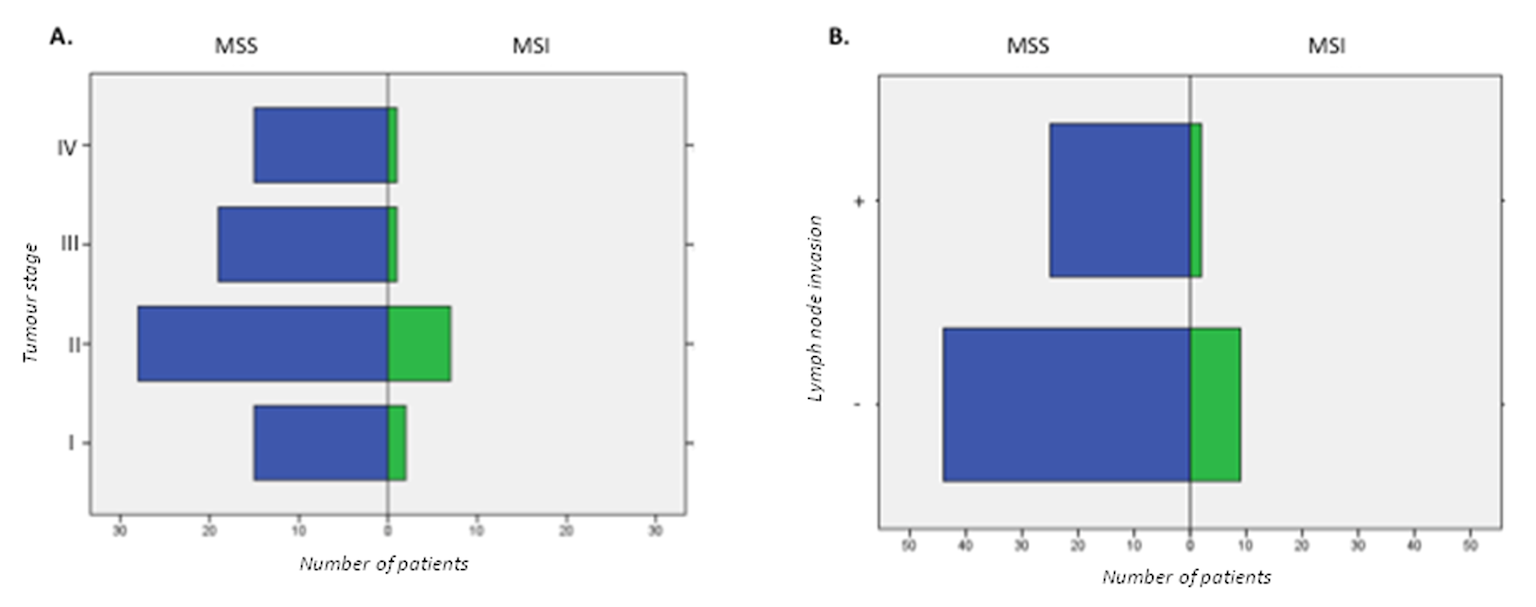

Supplement: S2 File — Tumour stage (Figure A) and lymphovascular invasion (Figure B) for microsatellite stable (MSS) versus instability (MSI) patients with colon cancer. (TIF) [file pone.0131421.s005.tif]

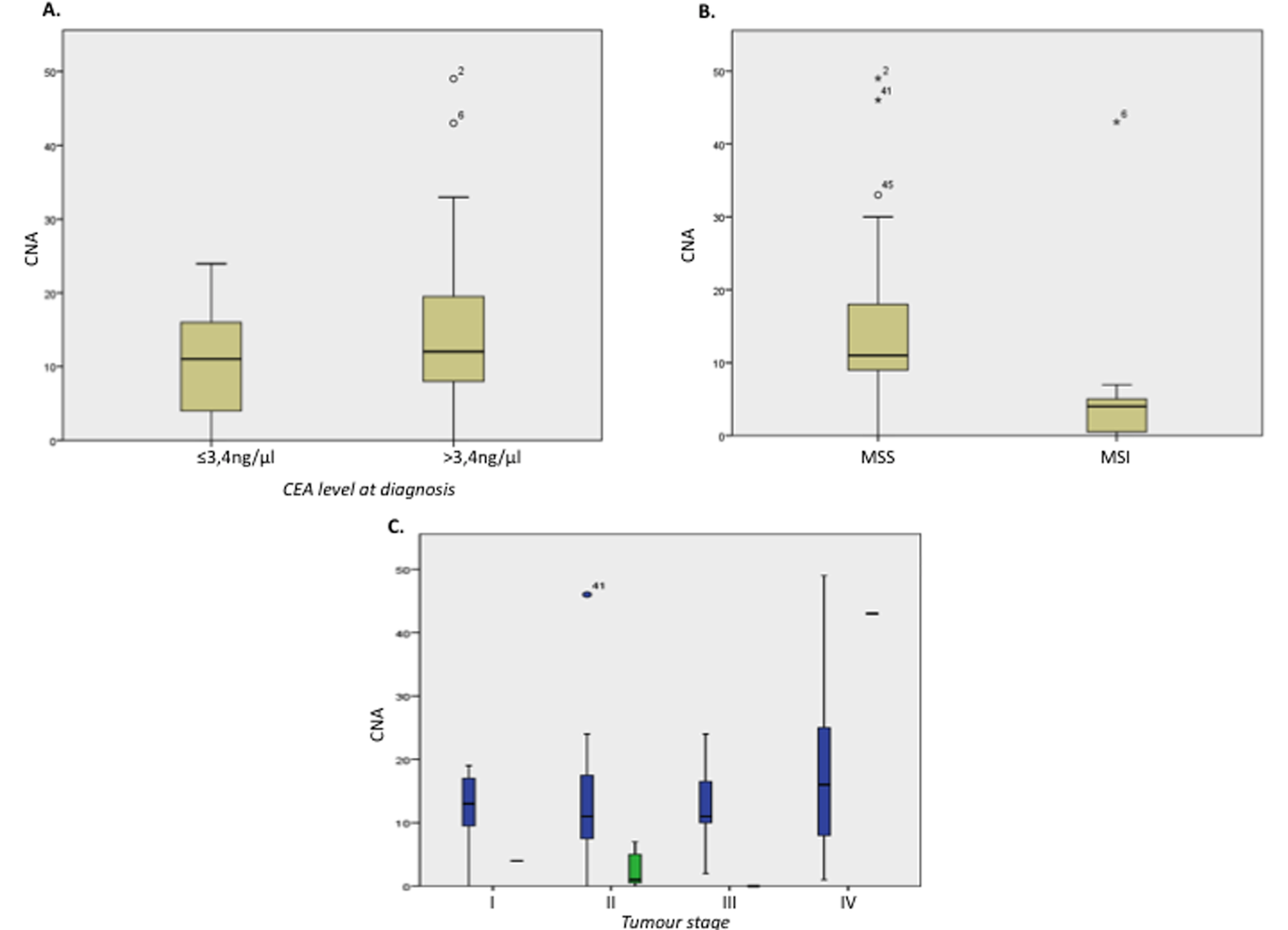

Supplement: S3 File — The CNAs are also grouped by MS status and tumour stage together (Figure C); with the MSS patients in blue and the MSI ones in green. (TIF) [file pone.0131421.s006.tif]

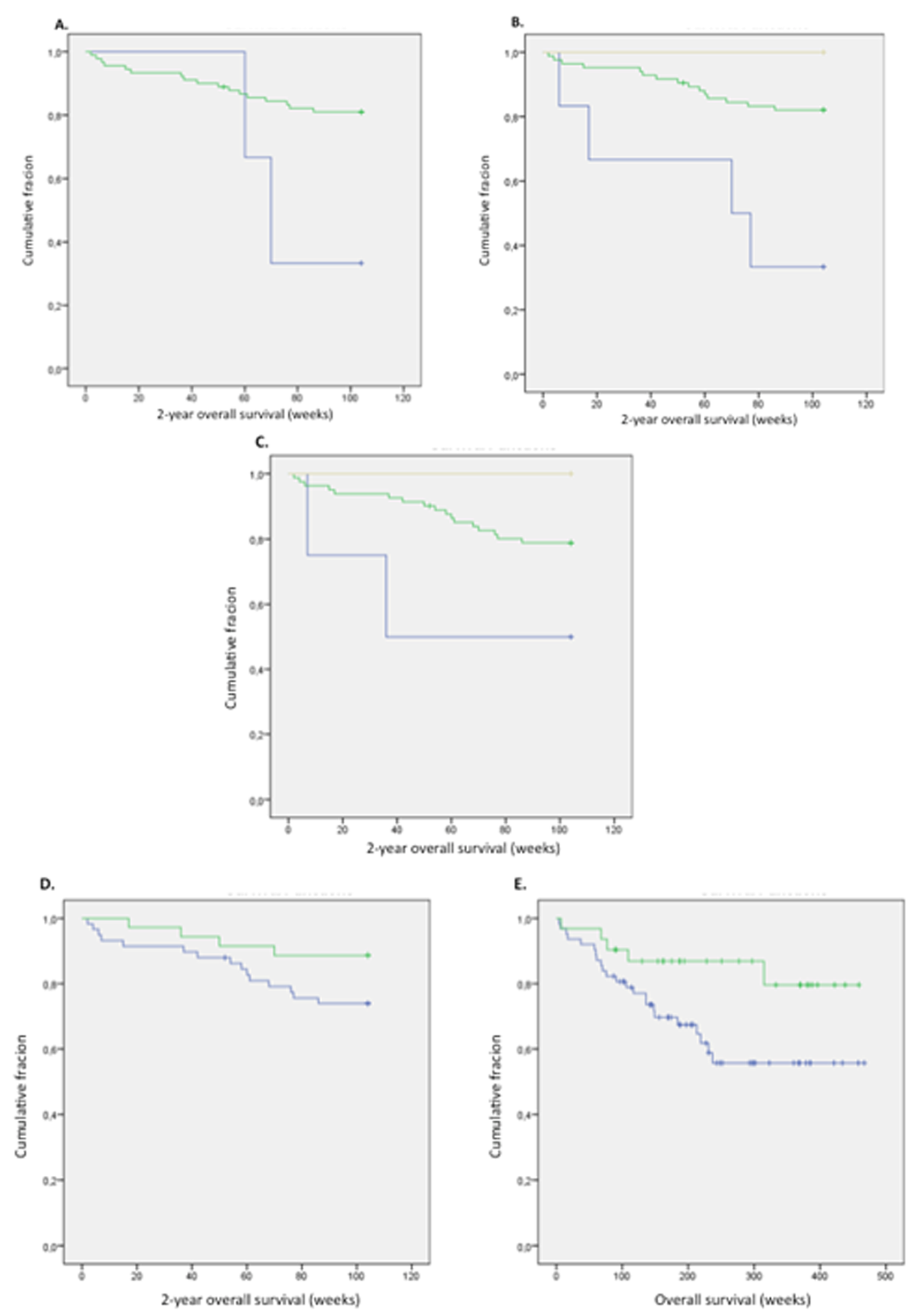

Supplement: S4 File — (Figure A) 2-year OS and chromosome 1 loss (blue = loss, green = no alteration); (Figure B) 2-year OS and chromosome 3 alteration (blue = loss, green = no alteration, yellow = gain); (Figure C) 2-year OS and chromosome 9 alteration (blue = loss, green = no alteration, yellow = gain); (Figure D) 2-year OS and chromosome 7 gain (blue = no alteration, green = gain); (Figure E) OS and chromosome 20 gain (blue = no alteration, green = gain). (TIF) [file pone.0131421.s007.tif]

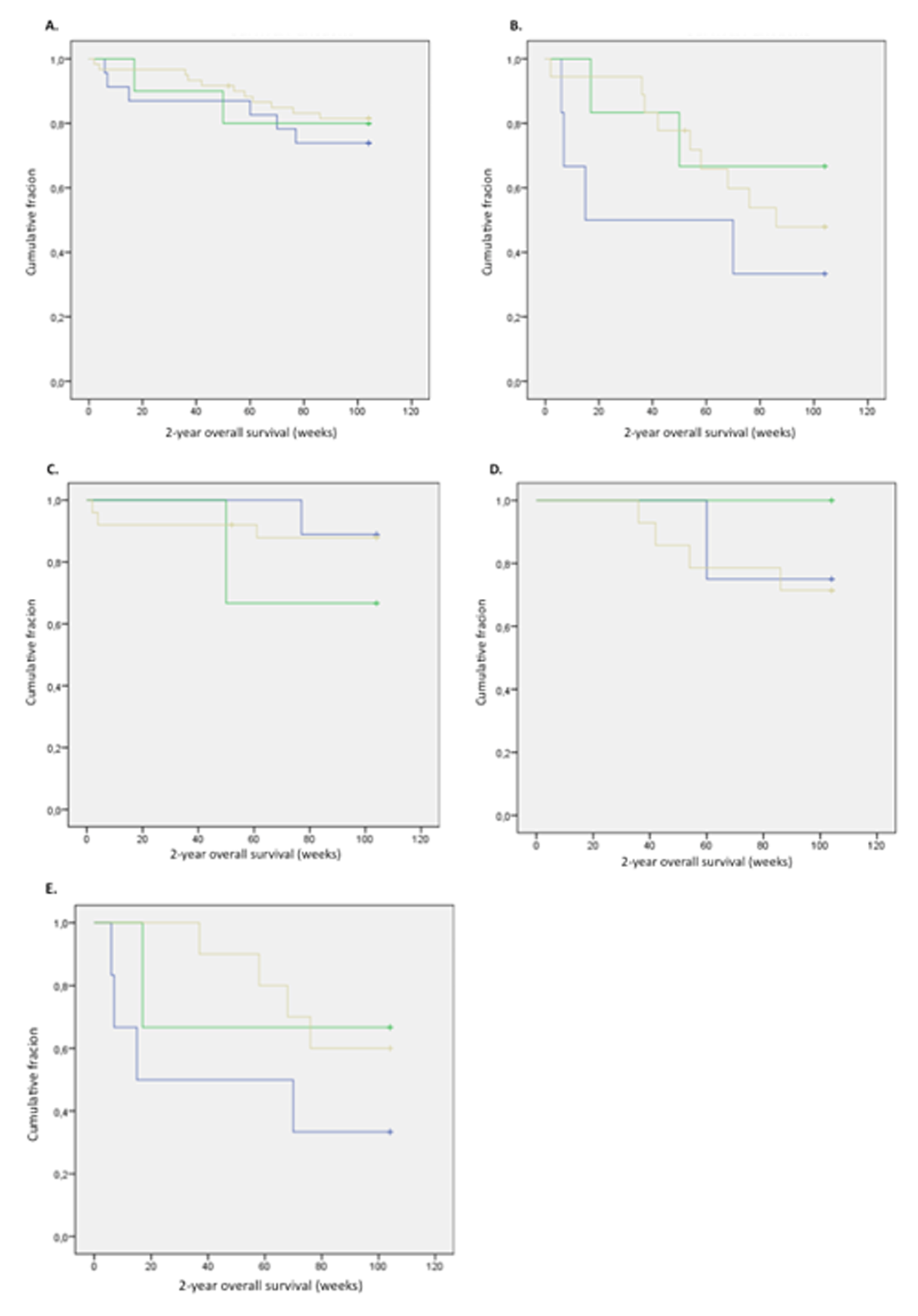

Supplement: S5 File — In blue, patients with a loss, in green, patients with a gain and in yellow patients without an alteration in the 16p13.3 locus. (TIF) [file pone.0131421.s008.tif]

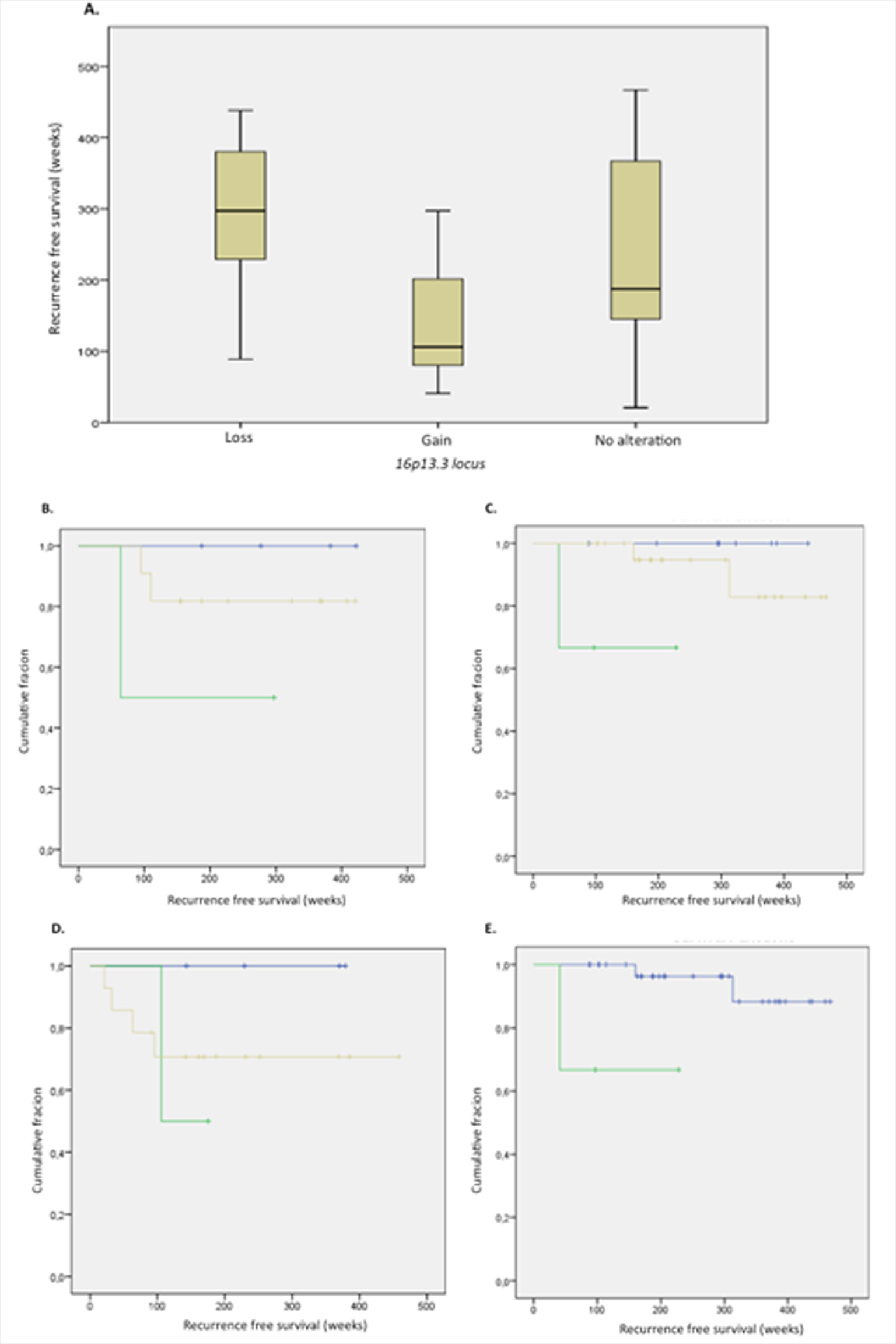

Supplement: S6 File — (Figure A) Boxplot for recurrence free survival in colon cancer and an alteration in the 16p13.3 locus (loss, gain or no alteration). Recurrence free survival (RFS) curves estimated by the Kaplan-Meier method for stage I (Figure B), stage II (Figure C), stage III (Figure D) and stage IV (Figure E) in colon cancer comparing with an alteration in the 16p13.3 locus. In blue, patients with a loss, in green, patients with a gain and in yellow patients without an alteration in the 16p13.3 locus. (TIF) [file pone.0131421.s009.tif]
