# Supplementary material for: Focus on 16p13.3 Locus in Colon Cancer
Source: PLoS One. 2015 Jul 29;10(7):e0131421. doi: 10.1371/journal.pone.0131421 (PMC4519182; doi:10.1371/journal.pone.0131421)
Supplement: S1 Table — (PDF) [file pone.0131421.s010.pdf]

Characteristics (n=159)	Number (percentage)
Gender	
Male	92 (57,9)
Female	67 (42,1)
Diabetes mellitus	
Yes	25 (15,7)
No	134 (84,3)
Obesity	
Yes	29 (18,2)
No	130 (81,8)
Tumour classification	
Adenocarcinoma	127 (79,9)
Mucinous carcinoma	32 (20,1)
Site of primary tumour	
Left	90 (56,6)
Right	69 (43,4)
Tumour stage: AJCC-7 classification	
I	29 (18,2)
II	58 (36,5)
III	39 (24,5)
IV	33 (20,8)
Differentiation grade*	
Well	11 (7,4)
Moderate	102 (68,5)
Poor	35 (23,5)
Non-differentiated	1 (0,7)
* 10 missing data	
CEA at diagnose*	
<3,4 ng/µl	61 (47,3)
>3,4 ng/µl	68 (52,7)
* 30 missing data	
MS status*	
MSS	131 (87,9)
MSI-low	2 (1,3)
MSI-high	16 (10,7)
* 10 missing data	
KRAS mutation*	
Wild-type	103 (66,9)
pGly12Ser	4 (2,6)
pGly12Asp	14 (9,1)
pGly12Val	14 (9,1)
pGly12Cys	7 (4,5)
pGly12Ala	2 (1,3)
pGly12Arg	2 (1,3)
pGly13Asp	8 (5,2)
* 5 missing data	
Perineural invasion (Pn)*	
Negative	104 (82,5)
Positive	22 (17,5)
* 33 missing data	
Lymphovascular invasion (Lv)*	
Negative	94 (66,2)
Positive	48 (33,8)
* 17 missing data	
Postoperative chemotherapy*	
None	79 (50,3)
Palliative	29 (18,5)
5-FU	17 (10,8)
Folfox	27 (17,2)
Capecitabin	5 (3,2)
* 2 missing data	
Recurrence	
Yes	18 (11,3)
No	111 (69,8)
Palliative	30 (18,9)
Survival status	
Death	54 (34)
Alive	105 (66)
