# Supplementary material for: Focus on 16p13.3 Locus in Colon Cancer
Source: PLoS One. 2015 Jul 29;10(7):e0131421. doi: 10.1371/journal.pone.0131421 (PMC4519182; doi:10.1371/journal.pone.0131421)
Supplement: S2 Table — (PDF) [file pone.0131421.s011.pdf]

	p-value	R
Gender <-> tumour stage	0,050	0,156
<-> postoperative chemotherapy	0,001	0,271
Diabetic <-> obesity	0,005	0,224
Age <-> 2-year OS	0,001	-0,258
<-> time to 2-year OS	0,003	0,234
<-> postoperative chemotherapy	<0,001	0,419
<-> site of primary tumour	0,001	0,259
<-> grade of differentiation	0,026	0,182
<-> MS status	0,029	-0,179
<-> Pn	0,022	0,204
<-> Lv	0,016	0,202
Obesity <-> recurrence	0,019	-0,185
<-> 2-year OS	0,039	-0,164
<-> time to 2-year OS	0,024	0,178
<-> tumour stage	0,021	-0,182
Classification <-> site of primary tumour	0,015	-0,193
<-> MS status	0,034	0,173
Site of primary tumour <-> recurrence	0,032	-0,171
<-> OS	0,001	-0,256
<-> MS status	<0,001	-0,291
Tumour stage <-> CEA at diagnosis	0,006	0,239
<-> Lv	<0,001	0,581
<-> Pn	<0,001	0,366
<-> resection status	<0,001	0,622
<-> time to OS	<0,001	-0,297
<-> postoperative chemotherapy	<0,001	0,570
<-> recurrence	<0,001	0,615
<-> OS	<0,001	0,322
<-> 2-year OS	<0,001	0,281
<-> time to 2-year OS	<0,001	-0,284
Differentiation grade <-> Type KRAS	0,027	0,184
<-> Adenome / Tumour	0,010	-0,209
<-> CEA at diagnosis	0,050	0,179
<-> Time to OS	0,031	-0,176
<-> 2-year OS	0,012	0,204
<-> time to 2-year OS	0,008	-0,216
CEA at diagnosis <-> resection status	<0,001	0,343
<-> time to recurrence	0,030	-0,191
<-> time to OS	<0,001	-0,372
<-> recurrence	0,003	0,260
<-> OS	0,001	0,288
<-> 2-year OS	0,002	0,271
<-> time to 2-year OS	0,001	-0,279
<-> postoperative chemotherapy	0,031	0,192
MS status <-> KRAS	0,017	-0,198
<-> Lv	0,038	-0,181
<-> type KRAS	0,031	-0,180
KRAS <-> Type KRAS	<0,001	0,961
<-> adenome / tumour	0,051	-0,157
Pn <-> Lv	<0,001	0,380
<-> resection status	0,001	0,286
<-> postoperative chemotherapy	0,006	0,246
<-> recurrence	0,001	0,296
<-> OS	0,054	0,172
Lv <-> resection status	<0,001	0,486
<-> postoperative chemotherapy	<0,001	0,363
<-> recurrence	<0,001	0,423
<-> OS	0,018	0,198
Resection status <-> recurrence	<0,001	0,655
<-> OS	<0,001	0,388
<-> time to OS	<0,001	-0,360
<-> 2-year OS	<0,001	0,296
<-> time to 2-year OS	<0,001	-0,314
<-> postoperative chemotherapy	0,019	0,187
Recurrence <-> time to recurrence	0,003	-0,236
<-> OS	<0,001	0,545
<-> time to OS	<0,001	-0,397
<-> postoperative chemotherapy	0,012	0,200
Time to survival <-> OS	<0,001	-0,571
<-> time to recurrence	<0,001	0,524
